# Efficacy of bottle gourd (*Lagenaria siceraria*) leaf extract in protecting against cabbage looper (*Trichoplusia ni)* (Lepidoptera: Noctuidae) infestation

**DOI:** 10.1007/s44297-025-00050-7

**Published:** 2025-05-23

**Authors:** Mst. Samia Sultana, Naoto Shimizu, Takanori Itoh, Kazunori Iwabuchi

**Affiliations:** 1 Graduate School of Agriculture, Hokkaido University, Hokkaido, 060-8589 Japan; 2 Research Faculty of Agriculture, Hokkaido University, Hokkaido, 060-8589 Japan

**Keywords:** Bottle gourd leaf, Phytochemical, Biopesticide, Plant extract, FTIR

## Abstract

Cabbage (*Brassica oleracea* L.) crops are frequently attacked by cabbage looper larvae (*Trichoplusia ni*), which severely reduce cabbage production. This experiment was conducted in the late spring crop season from April 15, 2024, to August 20, 2024, at the Field Science Center for the Northern Biosphere, Hokkaido University, Japan. In this study, the efficacy of an ethanolic extract of bottle gourd (*Lagenaria siceraria* (Molina) Standl.) leaves was compared with those of the chemical insecticide permethrin 3.2 EC and aqueous dimethyl sulfoxide (DMSO; the control). The experiment was carried out using a randomized complete block design in which each treatment comprised three replications consisting of 24 plants in total. Compared with the control, the bottle gourd leaf extract had high biopesticide activity against cabbage looper, increasing the cabbage yield by 15.98%. The yield was almost equal to that achieved with Permethrin 3.2 EC. Compared with the control, the application of the extract significantly decreased looper larval infestation in field-grown cabbage by 41.18%, 39.71%, 52.08%, and 37.96% at the fifth, sixth, seventh, and eighth weeks, respectively. Five possible biopesticide compounds, namely, apigenin-7-O-glucoside, indole-3-butyric acid, strychnine, phytol and hexadecanoic acid, were identified in the ethanolic extracts of bottle gourd leaves by liquid chromatography–mass spectrometry analysis. Bottle gourd leaf extract has potential as an environmentally friendly and sustainable alternative to toxic chemical pesticides for controlling cabbage looper infestation.

## Introduction

The use of synthetic chemical pesticides, which have various adverse effects on human health and the environment, must be reduced or prohibited to establish sustainable agricultural systems. Synthetic pesticides are dangerous when used frequently because they disrupt natural predators, pollinators, and other wildlife; contaminate groundwater; promote the development of resistance; and may cause the spread of secondary pests [1].

As ecofriendly biopesticides do not pollute the air, water, or soil with harmful chemicals, they are safer for the environment and human health. Botanicals are plant-derived substances that act via a variety of mechanisms [2]. These substances are derived from either fresh or dried plant materials, such as leaves, bark, flowers, roots, rhizomes, bulbs, seeds, cloves, or fruit [3]. When applied at the optimal time, concentration, and frequency, biopesticides can outperform synthetic pesticides [4]. Because biopesticides have a very short preharvest withholding period, they are safe for use on fresh fruits and vegetables [5].

Cabbage (*Brassica oleracea*) is a leafy green biennial plant known for its tightly packed heads of leaves and is a member of the Brassicaceae family [6]. Among the most widely used food crops, cabbage thrives in many regions of the world [7]. In 2022, total cabbage production in Japan was 1,458,200 Mt, according to Japan’s Ministry of Agriculture, Forestry and Fisheries. Cabbage plants can be infested by many types of insects, such as cabbage looper, aphids, large white butterfly, diamondback moth, cabbage moth, cabbage maggots, thrips, and striped flea beetles, depending on the variety of cabbage, weather, and region. During the present experiment, only the cabbage looper (*Trichoplusia ni*) was visible from the seedling stage to the final growth stage. Other pests were present in extremely minor quantities and thus were not taken into consideration.

The cabbage looper larva is a type of cabbage worm, which is a general term for insects in the Lepidoptera order that primarily feed on cruciferous vegetables. The cabbage looper is most problematic once the plant enters the heading stage. The larvae eat the underside of the leaves and consume the developing head. The infamous reputation of this pest likely stems from its ability to infest a variety of crops readily and the difficulty in managing the pest [8]. Eggs are deposited singly or in small clusters on either leaf surface, although more eggs are found on the lower leaf surface. Each female moth can produce 300–600 eggs within approximately 10–12 days. After the eggs hatch, additional larvae move to the lower leaf surface to feed. Two to four weeks after hatching, the mature larva forms a thin cocoon on the lower leaf surface or in plant debris or soil. The pupal stage lasts approximately two weeks. With warm temperatures, the development of cabbage looper, from egg to adult, takes approximately 18 to 25 days [9].

Bottle gourd (*Lagenaria siceraria*), also called white-flowered gourd or calabash gourd, is a running or climbing vine of the gourd family (Cucurbitaceae). Bottle gourd was among the first plants to be domesticated and the only one with a global distribution during pre-Columbian times [10]. Bottle gourd leaf extracts, at 10% and 20% concentrations, have shown good efficacy in reducing aphid populations on cabbage plants [11]. Islam et al. reported that a water gourd leaf extract showed good efficacy against aphids in a cabbage field experiment [12]. A petroleum ether extract from the leaves of *L. siceraria* provided maximum protection (100%) against *Culex pipiens* larvae [13]. Thus, bottle gourd leaf extracts may be useful as biopesticides in cabbage fields instead of as chemical pesticides to control cabbage looper infestations and reduce environmental pollution. Therefore, the present study was conducted to determine the efficacy of a bottle gourd leaf extract in controlling cabbage looper growth in field-grown cabbage, to determine the phytochemical effects of the extract on the growth and yield of cabbage, and to analyze the phytochemical composition of the bottle gourd leaf.

## Materials and methods

### Experimental site and plot preparation

This experiment was conducted in the late spring crop season from April 15, 2024, to August 20, 2024, at the Field Science Center for the Northern Biosphere, Hokkaido University, Hokkaido, Japan. The experimental site is located at 43°4ʹ N latitude and 141°20ʹ E longitude at an altitude of 20 m above sea level. The weather conditions from seedling to harvest are listed in Table S1, and the characteristics of the soil properties present in the cultivation conditions are listed in Table S2. The experiment was carried out in a randomized complete block design, with each treatment comprising three replications consisting of 24 plants in total. The unit plot size was 1.2 m × 1.8 m, the distance between the blocks (i.e., between replications) was 0.75 m, the spacing between individual plants was 0.45 m, and the spacing between rows was 0.5 m. The soil was prepared for sowing by ploughing it five times, followed by laddering. The crop stubble and uprooted weeds were removed from the soil, and the land was leveled before the cabbage seedlings were planted. Before the seedlings were transplanted, 25 g of organic fertilizer was incorporated into every planting hole.

The determinate cabbage variety used in this experiment, ‘Kinkei-201’, is popular with local growers and was purchased from Snow Brand Seed Co., Ltd. (Sapporo, Hokkaido, Japan). At the four-leaf stage, uniform healthy seedlings were transplanted into the experimental plots (Fig. S1). The field was irrigated a total of 12 times during the growing season. Field management for protection from small animals was achieved by installing two area pole insulators Ø 20 AP-PL900GB, an Apollo Solar Electric Fence Area System body AP-2011-SR, and a 500 m orange Shinsei electric fence rope.

### Preparation of treatment and control solutions

Bottle gourd leaves were collected from the campus of Hokkaido University, Japan, as a source of plant materials with potential biopesticide properties. The samples were first visually examined for any type of infection, spores, damage, discolouration, or distortion. Undamaged leaf samples were thoroughly washed with running tap water and then allowed to dry in ambient light at room temperature. The dried leaves were ground to powder with a high-speed grinder (WB-1, Osaka Chemical Co., Ltd., Osaka, Japan). Approximately 100 g of the powder was placed in a conical flask, and 400 ml of ethanol was added. The conical flasks were shaken in a constant-temperature incubator shaker (Bioshaker BR-41 FL, TAITEC Co., Koshigaya City, Saitama, Japan) for 74 h at 120 rpm. After centrifugation (Tomy MX-305 high-speed refrigerated microcentrifuge, Tomy Kogyo Co., Ltd., Fukushima, Japan) for 20 min at 2075 × *g*, the extract was filtered through Whatman No. 1 filter paper. The filtrate was placed in an EYELA-type constant-temperature air-drying oven (WFO-420 W, Tokyo Rikakikai Co., Ltd., Tokyo, Japan) at 50 °C to evaporate the ethanol. Then, 1 g of crude extract from the bottle gourd leaves was dissolved in 1 ml of DMSO and added to 1000 ml of distilled water. The trial testing the efficacy of the crude extract also included a control (0.1% [v/v] aqueous DMSO) and a chemical insecticide treatment, permethrin (3.2 emulsifiable concentrate), as the positive control. The positive control, permethrin (3.2 EC formulation), was applied at a rate of 10 mL per 1 L of water (1% [v/v] aqueous solution). The concentrations of the treatment and control solutions are shown in Table 1. Permethrin is a widely used pesticide owing to its effectiveness against insects and its low toxicity to mammals. Permethrin acts on the nerve cell membrane to disrupt the sodium channel current. Delayed repolarization and paralysis of pests result from this disturbance [14]. DMSO (CH_3_)_2_SO is a powerful solvent for a wide range of organic and inorganic compounds, is suitable for diluting plant extracts that may contain a mixture of different compounds, and concentrations of less than 0.1% are safe for plants and animals [15].

**Table 1:** Concentrations of the treatment and control solutions

Treatments	Chemical pesticide (Permethrin 3.2 EC)	Bottle gourd leaves	Control (aqueous DMSO)
Concentrations	1%	0.1%	0.1%

### Spray application of solutions

The prepared solutions were sprayed onto the cabbage seedlings in the experimental field with a hand-held garden sprayer (capacity 1.5 L; Taizhou City Hangyu Plastic Co., Ltd., Zhejiang, China) in the morning (09:00) at 7-day intervals. The spray applications were initiated at 10 days after transplanting (DAT) the cabbage seedlings and continued until the end of the growing season (58 DAT). During this growing period, cabbage plants were sprayed eight times in total with the treatments and the control at a volume of 10 ml per cabbage plant per application.

### Looper infestation monitoring and growth indicator measurement

Cabbage looper infestations on the cabbage plants were monitored from the start of insect colonization until the cabbage plants reached maturity. First, the number of leaf perforations on all infested leaves was counted with the naked eye (Fig. S2). The number of insects was counted using a per-leaf sampling system [16]. The plots were sampled daily by visual inspection of both leaf surfaces in the morning for 7 weeks from July 10 to August 30. Cabbage looper larvae within the canopy were counted for eight cabbage plants in each plot. These larvae were collected by hand from each treatment plot and placed in a plastic bag for disposal. Plant growth indicators were measured in the field in August 2024, and yield data were collected after harvesting at 70 DAT. The plant height and stem diameter (cm) were measured, and the SPAD value was determined to estimate the leaf chlorophyll content, which was measured with a portable chlorophyll meter (SPAD-502, Minolta Camera Co., Ltd., Osaka, Japan). The photosynthesis rate was measured with a plant photosynthesis meter (miniPPM-300, EARS, Delft, The Netherlands). The total cabbage yield (kg/plot) was recorded, and the percentage yield increase over that of the control was calculated as follows [17]:

$$\text{Yield increase or decrease }({\%})=\frac{\text{Treatment yield}-\text{Control yield}}{\text{Control yield}}\times100$$

The percentage of cabbage looper infestation (%) in the bottle gourd leaf extract group was calculated as follows:

$$\text{Cabbage looper infestation }({\%}) =\frac{\text{Looper number in bottle gourd leaf extract group}-\text{Looper number in Control group}}{\text{Looper number in Control group}}\times 100$$

### Sample preparation for phytochemical analysis

Bottle gourd leaf extract preparation was performed following standard procedures described previously. All the chemicals and solvents used for LC–MS were of HPLC grade.

### Liquid chromatography–mass spectrometry analysis

Liquid chromatography–mass spectrometry (LC–MS) analysis of the ethanolic extract of bottle gourd leaves was performed using a Nexera-XR liquid chromatograph (Shimadzu, Kyoto, Japan) coupled with a mass spectrometer (LTQ-Orbitrap XL, Thermo Fisher Scientific, Waltham, MA, USA). The extract (5 µL) was injected into an Inert Sustain AQ-C18 column (1.9 µm, 2.1 × 100 mm; GL Sciences, Tokyo, Japan) maintained at 40 °C. Aqueous formic acid (0.1%, solvent A) and 0.1% formic acid in acetonitrile (solvent B) were used as the mobile phases. Gradient elution was carried out at 150 µL/min using the following software: 50% B for 0 min, 95% B for 15 min, 95% B for 20 min, 50% B for 21 min, and 50% B for 26 min.

Electrospray ionization was used in positive mode. Other instrument settings were as follows: source voltage, 4.0 kV; nitrogen sheath gas flow rate, 30 L/min; auxiliary gas flow rate, 10 L/min; sweep gas flow rate, 0 L/min; capillary temperature, 300 °C; capillary voltage, 20 V; and tube lens voltage, 80 V. All extracts were evaluated in Fourier transform mass spectrometry (FTMS) mode, and the mass range was acquired by full-range acquisition covering *m*/*z* 100–2000. The samples were typically diluted (1:200) with solvent A. Data analysis was performed using XCalibur software v2.0.7 (Thermo Fisher Scientific).

### Fourier transform infrared spectroscopy analysis

The functional groups and molecular structures on the surface of the ethanolic extracts of bottle gourd leaves were examined using Fourier transform infrared (FTIR) spectroscopy (FT/IR-4X, JASCO Corporation, Tokyo, Japan). Each spectrum was produced by averaging 50 scans in the range of 500 − 4000 cm^−1^ with a resolution of 4 cm^−1^. The baselines were adjusted for atmospheric CO_2_ and water vapour to ensure accurate readings.

### Statistical analysis

The biopesticide activity of bottle gourd leaf extract against cabbage looper larvae on field-grown cabbage plants was tested in an experimental plot using a randomized complete block design. The data were entered into Excel (Microsoft) and subjected to one-way analysis of variance. Significant differences among treatments were detected using Duncan’s multiple range test (*P* < 0.05) using IBM SPSS Statistics v.20 software (IBM Corp., Armonk, NY, USA) [18].

## Results

### Effects of ethanolic extracts of bottle gourd leaves on the average number of cabbage looper larvae per plant at the fifth, sixth, seventh and eighth weeks

**Fig. 1:**
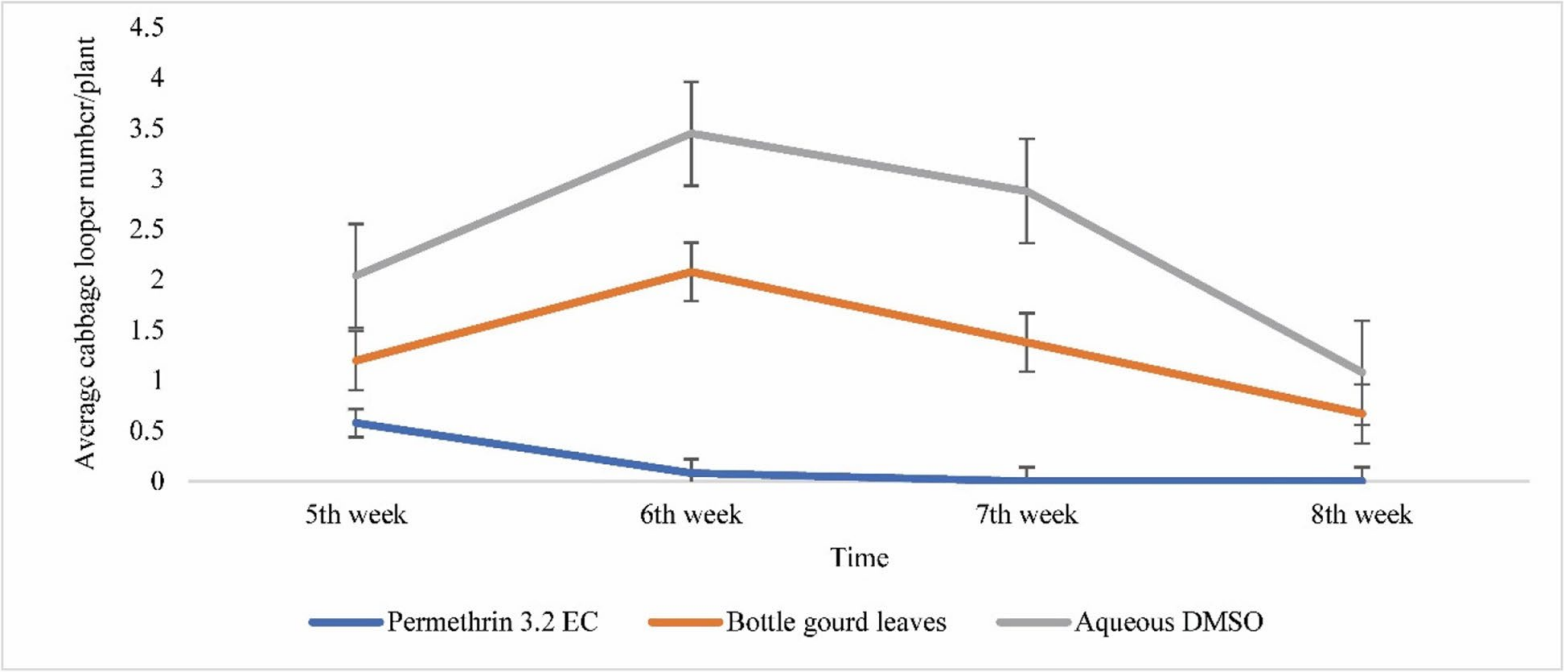
Biopesticide activity of the ethanolic extract of bottle gourd leaves against cabbage looper (*Trichoplusia ni*) larvae relative to that of permethrin and aqueous DMSO. The error bars represent the standard errors (*P* < 0.05; *n* = 24)

The chemical pesticide permethrin provided the strongest protection against cabbage looper larvae (Fig. 1). Compared with the control (aqueous DMSO), the bottle gourd leaf extract had good efficacy against cabbage looper larvae. Compared with the control (aqueous DMSO), the application of the extract significantly decreased looper larval infestation by 41.18%, 39.71%, 52.08%, and 37.96% at the fifth, sixth, seventh, and eighth weeks, respectively, in field-grown cabbage. The greatest infestation of cabbage looper larvae was observed at the sixth week, whereas the infestation at the fifth and seventh weeks was low, and after the eighth week, no larvae were observed.

### Effects of the ethanolic extract of bottle gourd leaves on the total number of leaves per plant, number of infested leaves, and average number of perforations per infested leaf at 30, 40, 50, and 60 DAT

**Fig. 2:**
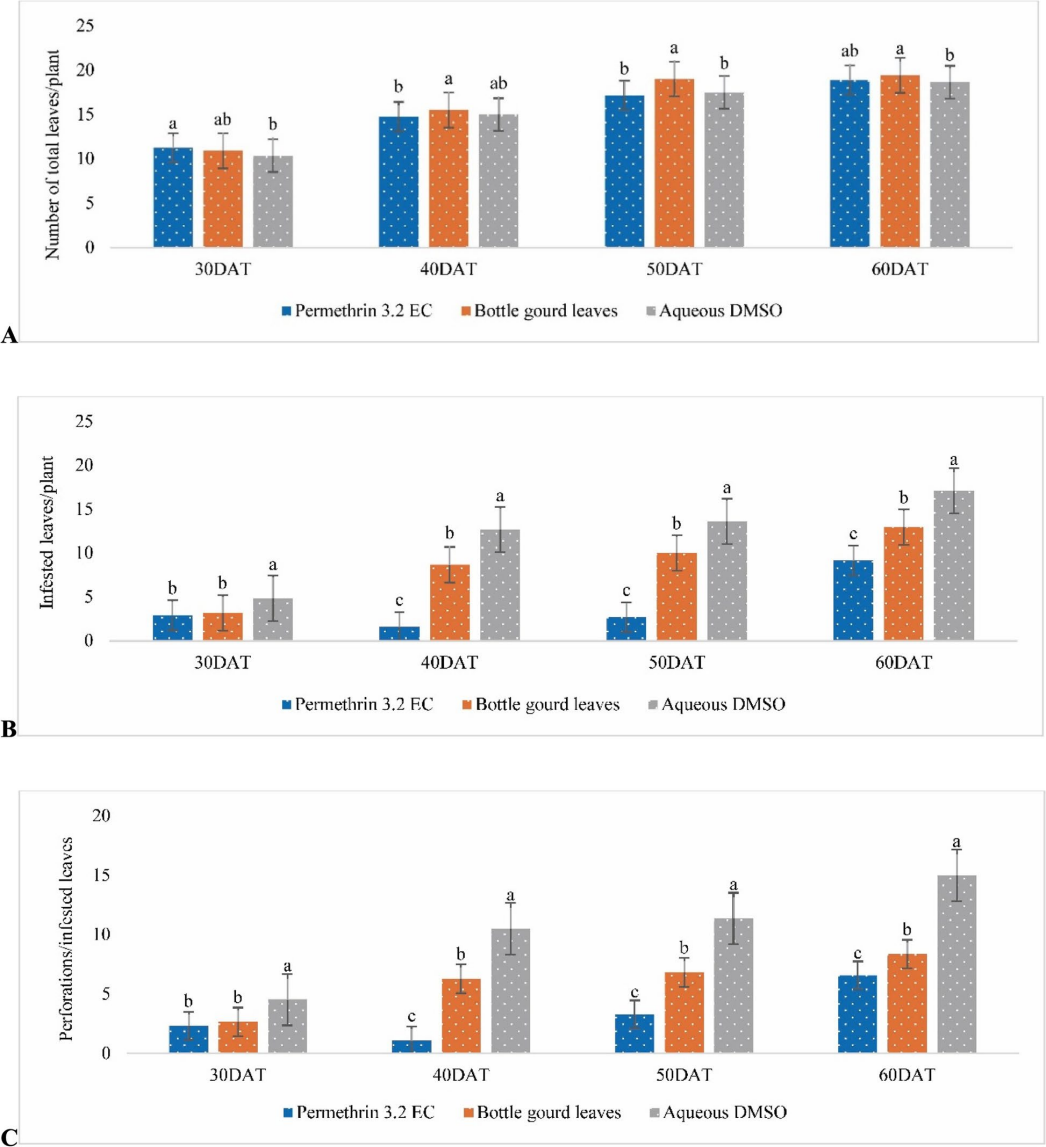
Effects of the ethanolic extract of bottle gourd leaves on the total number of leaves per plant; **B** infested leaves, and **C** average perforations per infested leaf of cabbage at 30, 40, 50, and 60 days after treatment (DAT). Different lowercase letters above the bars indicate significant differences as assessed by Duncan’s multiple range test, and the error bars represent the standard error (*P* < 0.05; *n* = 24)

Cabbage plants in the treatment groups and the control group produced very similar total numbers of leaves (Fig. 2A). The number of infested leaves was very low in response to the chemical pesticide treatment, whereas the bottle gourd leaf extract had a moderate effect compared with that of the control (Fig. 2B). The number of perforations in infested leaves of cabbage plants treated with the bottle gourd leaf extract was moderate at 40 and 50 DAT, but at 60 DAT, the number of perforations was similarly reduced by the bottle gourd leaf extract and the chemical pesticide compared with the control (Fig. 2C).

### Effects of the ethanolic extract of bottle gourd leaves on the relative chlorophyll content (SPAD), photosynthesis rate, and plant growth indicators

**Fig. 3:**
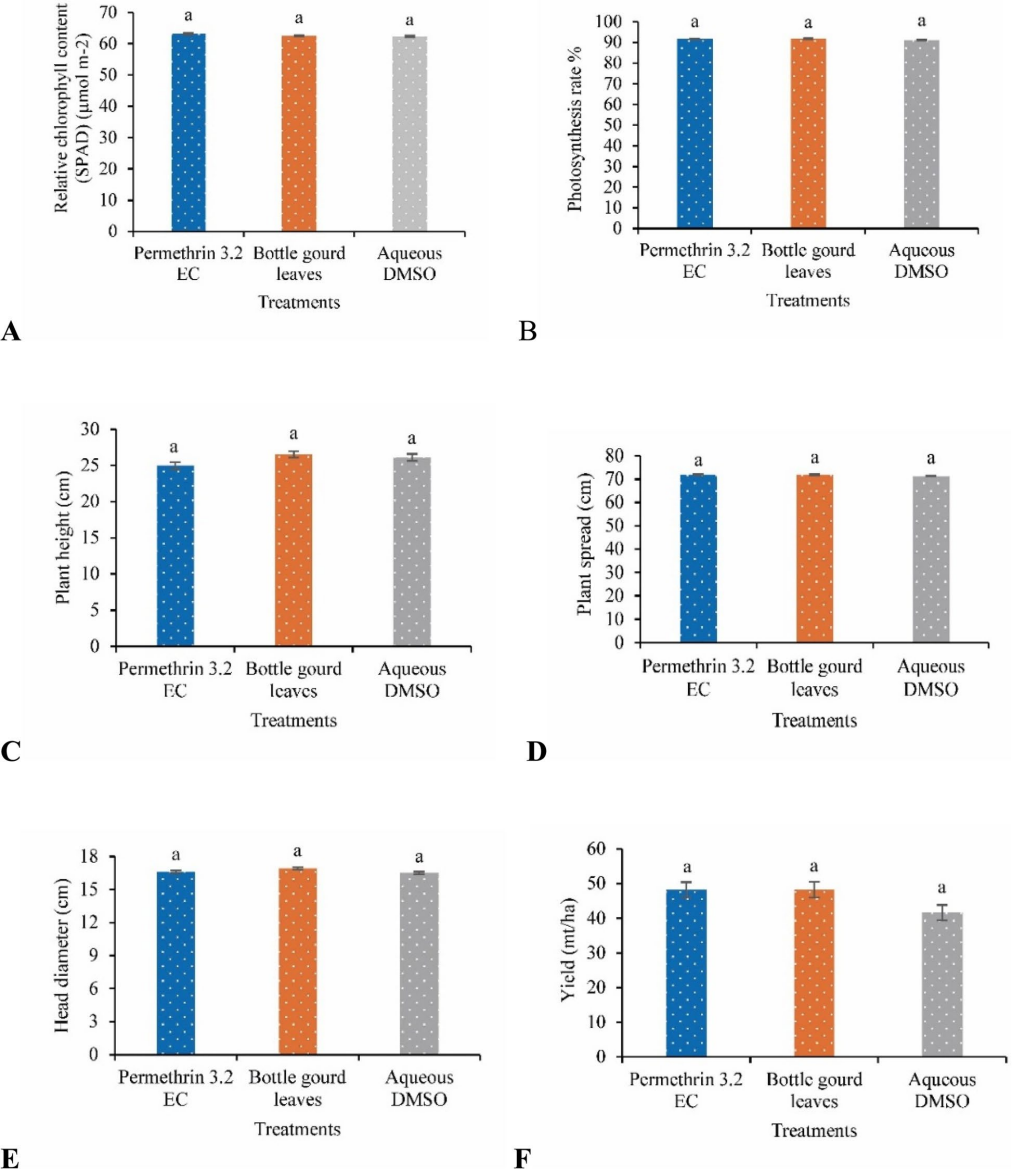
Effects of the ethanolic extract of bottle gourd leaves on the **A** relative chlorophyll content (SPAD); **B** photosynthesis rate; **C** plant height; **D** plant spread; **E** head diameter; and **F** yield. Different lowercase letters above the bars indicate significant differences as assessed by Duncan’s multiple range test, and error bars represent standard errors (*P* < 0.05; *n* = 24)

No significant difference in the relative chlorophyll content (SPAD) or photosynthesis rate was detected between the permethrin 3.2 EC and bottle gourd leaf extract treatments and the control (Fig. 3A and B). Compared with the chemical pesticide and control groups, the group treated with the bottle gourd leaf extract presented the greatest plant height, although the difference was not significant (Fig. 3C). No significant differences in plant spread (cm) or head diameter (cm) were detected among the permethrin 3.2 EC and bottle gourd leaf extract treatments compared with the control (Fig. 3D and E).

### Effects of the ethanolic extract of bottle gourd leaves on cabbage yield

The chemical pesticide and bottle gourd leaf extract treatments produced yields of 48.23 and 48.26 mt/ha cabbage, respectively, and provided good protection from cabbage looper larvae. In contrast, the control (aqueous DMSO) produced 41.61 mt/ha, and the efficacy against looper infestation was very poor. The bottle gourd leaf extract showed high biopesticide activity against cabbage looper infestation and increased the cabbage yield by 15.98% compared with that of the control (aqueous DMSO), which was almost equal to that of the permethrin 3.2 EC-treated group (Fig. 3F).

### Characterization and identification of biopesticide compounds in the ethanolic extract of bottle gourd leaves by LC–MS analysis

LC–MS analysis of the ethanolic extract of bottle gourd leaves enabled the identification of 21 chemical compounds (Fig. S3): apigenin-7-O-glucoside; 4-isopropyl-2-nitrophenol; 1-myristoyllysophosphatidic acid; (2,2-dichloro-1-(4-methyl-benzoylamino)-vinyl)- phosphonic acid diethyl ester; pentafluorobenzoic acid, 2-amino-9H-pyrido[2,3-b]indole; succinic acid, 2,2,3,3,4,4,5,5-octafluoropentyl pentafluorobenzyl ester; lyalosidic acid; indole-3-butyric acid; 5-(3,4,5-trimethoxybenzoyl)−2,4-pyrimidinediamine; phenol, 2-methoxy-4-(1-propenyl)-, acetate, (Z)-; strychnine; cytosine, N,N'-di(pentafluoropropionyl)-; phytol; 2-methyl-1,4-butanediol, bis(heptafluorobutyrate); 3-hydroxyechinenone; 7-acetyl-1,dione;(1Z)−1-[(3,4-diethoxyphenyl)methylidene]−6,7-diethoxy-3,4-dihydro- Basic information on each component of the bottle gourd leaf extract is presented in Table 2.

**Table 2:** Tentatively identified compounds in the ethanolic extract of bottle gourd leaves and their respective retention times (R/T), chemical names, elemental formulas, detected *m*/*z values*, and exact *m*/*z values*

No.	R/T	Proposed compounds	Elemental formula	Detected *m/z*	Exact *m/z*	% of composition
1	1.85	Apigenin-7-O-glucoside	C_21_H_20_O_10_	433.1126	432.1057	8.65
2	2.13	4-isopropyl-2-nitrophenol	C_9_H_11_NO_3_	182.9850	181.002	3.24
3	2.99	1-Myristoyllysophosphatidic acid	C_17_H_35_O_7_P	383.1513	382.436	3.24
4	5.74	(2,2-Dichloro-1-(4-methyl-benzoylamino)-vinyl)- Phosphonic acid diethyl ester	C_14_H_18_Cl_2_NO_4_P	367.1506	366.184	9.73
5	6.76	Pentafluorobenzoic acid, pent-2-en-4-ynyl ester	C_12_H_5_F_5_O_2_	277.2163	276.1589	4.87
6	7.53	2-Amino-9H-pyrido[2,3-b]indole	C_11_H_9_N_3_	184.9857	183.21	10.81
7	8.57	Succinic acid, 2,2,3,3,4,4,5,5-octafluoropentyl pentafluorobenzyl ester	C_16_H_9_F_13_O_4_	513.2529	512.2195	2.16
8	8.74	Indolebutyric acid	C_12_H_13_NO_2_	205.1958	203.2371	8.65
9	10.05	5-(3,4,5-Trimethoxybenzoyl)−2,4-pyrimidinediamine	C_14_H_16_N_4_O_4_	305.2479	304.1172	1.08
10	10.55	Phenol, 2-methoxy-4-(1-propenyl)-, acetate, (Z)-	C_12_H_14_O_3_	207.1033	206.2378	10.27
11	10.75	Strychnine	C_21_H_22_N_2_O_2_	335.1520	334.1681	2.16
12	12.69	Cytosine, N,N'-di(pentafluoropropionyl)-	C_10_H_3_F_10_N_3_O_3_	404.3161	403.1332	1.08
13	13.25	Phytol	C_20_H_40_O	297.3161	296.53	10.27
14	15.3	2-Methyl-1,4-butanediol, bis(heptafluorobutyrate)	C_13_H_10_F_14_O_4_	497.2578	496.1937	1.08
15	15.35	3-Hydroxyechinenone	C_40_H_54_O_2_	568.4281	566.9	1.08
16	15.70	7-Acetyl-1,3-dimethylpurine-2,6-dione;(1Z)−1-[(3,4-diethoxyphenyl)methylidene]−6,7-diethoxy-3,4-dihydro-2H-isoquinoline	C_33_H_41_N_5_O_7_	621.3080	619.7	6.49
17	17.56	PE 34:3	C_39_H_72_NO_8_P	714.5533	713.4996	1.08
18	17.75	Hexadecanoic acid	C_18_H_36_O_2_	285.2788	284.4772	10.27
19	20.82	Luteolin 7-glucuronide-3',4'-dirhamnoside	C_33_H_38_O_20_	756.5566	754.647	0.54
20	23.27	Ciprofloxacin	C_17_H_18_FN_3_O_3_	332.9163	331.347	0.54
21	24.34	2,6-Dimethylquinoline	C_11_H_11_N	158.9615	157.21	2.70

A literature search revealed that, among these constituents, apigenin-7-O-glucoside, indole-3-butyric acid, strychnine, phytol and hexadecanoic acid are highly effective in the management of cabbage looper

**Fig. 4:**
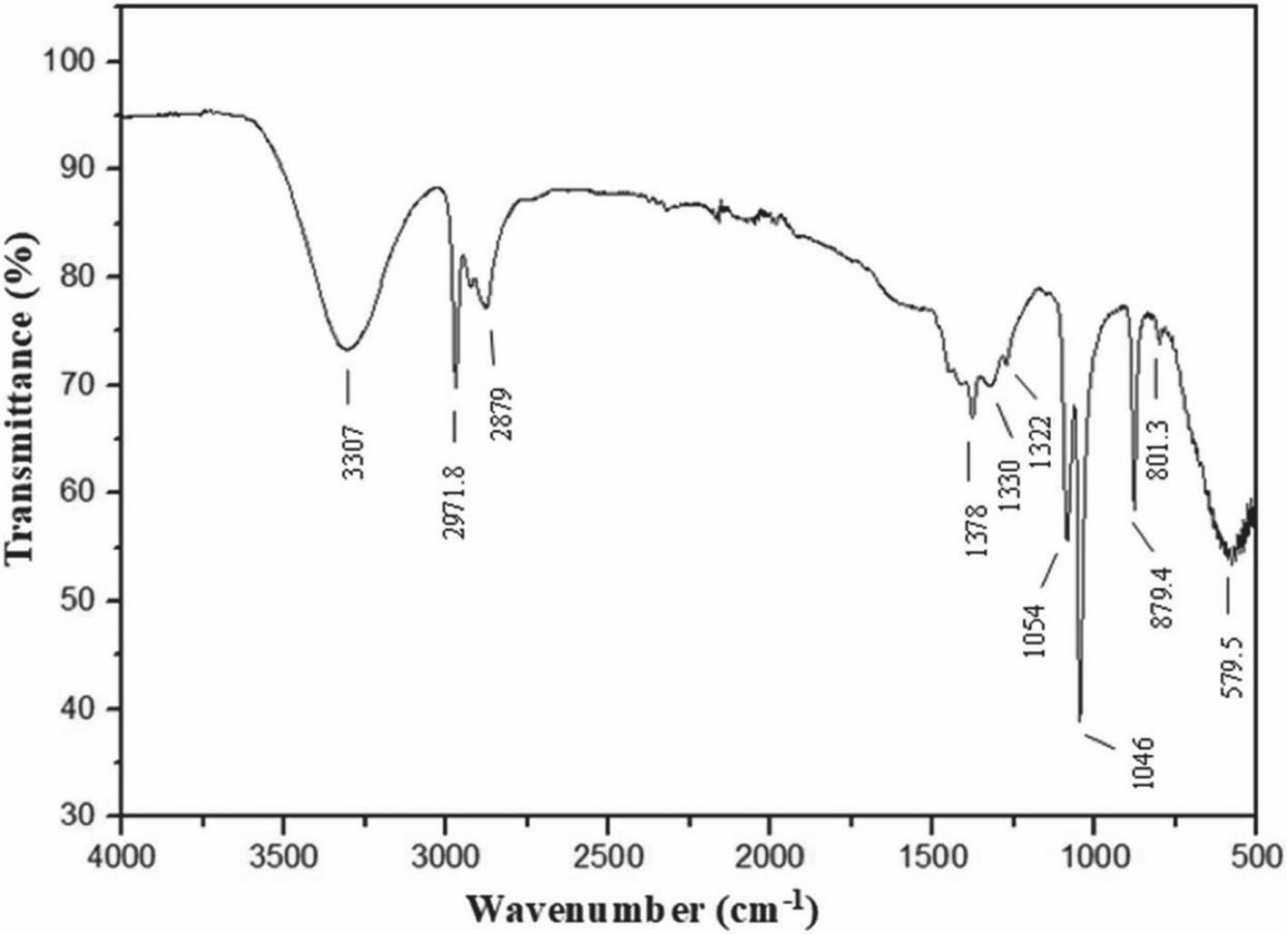
Fourier transform infrared spectroscopy spectrum of the ethanolic extract of bottle gourd leaves

FTIR analysis reveals the infrared spectrum of a material by collecting high-resolution spectral data over a specific spectral range and displays the extent of absorption of a single wavelength of infrared light by a sample at each wavelength [19]. The present FTIR analysis revealed absorption bands at 3307, 2971.8, 2879, 1378, 1330, 1322, 1054, 1046, 879.4, 801.3, and 579.5 cm^−1^, indicating the presence of capping or stabilizing agents in the ethanolic extracts of bottle gourd leaves (Fig. 4). The intense band at 3307 cm-1 corresponded to N–H stretching, which confirmed the presence of a primary aliphatic amine. The band at 2971.8 cm^−1^ corresponded to O–H stretching, confirming the presence of carboxylic acid. The peak at 2879 cm^−1^ corresponded to C–H stretching and confirmed the presence of alkanes. The peak at 1378 cm^−1^ was attributed to O–H bending and confirmed the presence of alcohol. The band observed at 1330 cm^−1^ was consistent with S = O stretching and indicated the presence of sulfone. The band at 1322 cm^−1^ corresponded to O–H bending and indicated the presence of phenol. The bands at 1054 and 1046 cm^−1^ were attributed to C-O stretching and CO–O-CO stretching, respectively, which indicated the presence of primary alcohol and anhydride, respectively. The bands at 879.4 and 801.3 cm^−1^ were attributed to C–H bending and C–C bending, respectively, which indicated the presence of a 1,2,4-disubstituted compound and an alkene, respectively. The 579.5 cm^−1^ band was consistent with C–Cl stretching and indicated the presence of a halo compound.

## Discussion

Strong links have been established between exposure to pesticides and increased risk of several chronic diseases, such as various types of cancers, neurological disorders, cardiovascular diseases, developmental delays in children, effects on reproductive capacity, male and female infertility, cognitive impairments, and impaired respiratory health. Recent assessments have shown that pesticide exposure is linked to a broad range of direct and indirect effects on biodiversity, contributing to declines in populations of insects, birds, bats, earthworms, aquatic plants, fishes, and amphibians, among other organisms [20].

Plant biopesticides are safe for both the applicant and the consumer because they are nontoxic [21]. In addition, they do not affect beneficial organisms, such as natural enemies of pests, because they are target specific [22]. Therefore, such substances can be effectively included in integrated pest management programs to decrease the volume of chemical pesticides used in the control of agricultural pests [23]. Furthermore, natural materials are safer to use in the environment because they decompose quickly [24].

The plant chemical defense system comprises secondary metabolites, including phenols, flavonoids, quinones, terpenes, tannins, alkaloids, lectins, polypeptides, saponins, and sterols [25]. Plant extracts rich in such secondary metabolites can disrupt cell membranes, inactivate a variety of enzymes, and disrupt the metabolic processes of insects, leading to growth inhibition or death. Plant extracts tend to have broad-spectrum activity, are relatively specific in their mode of action, and are easy to process and use at the farm level [26].

The cabbage looper is a medium-sized moth in the family Noctuidae, a family that is frequently called"owlet moths."The larvae eat large holes on the underside of the leaves and consume developing cabbage heads. Larvae initially do not consume much food but increase their consumption during their lifetime until they are consuming three times their weight daily [27].

Bottle gourd is an annual herbaceous plant with a prostrate growth habit [28]. It is planted worldwide because of its high nutritional and medicinal properties [29]. Ethnobotanical, epidemiological, and traditional information associated with the medicinal and nutritional capacity of bottle gourd has been thoroughly reviewed [30]. A bottle gourd leaf extract shows larvicidal activity against third-instar larvae of *Musca domestica* [31]. A bottle gourd extract has been used as a natural insecticide against the mustard aphid *Lipaphis erysimi* [32]. Like other members of the Cucurbitaceae, gourds contain cucurbitacins, which are known to be cytotoxic at high concentrations [33]. Aldewy et al. tentatively identified 14 hexane-soluble compounds from air-dried leaves of bottle gourd by gas chromatography − mass spectrometry [34].

In this study, the efficacy of a bottle gourd leaf extract was compared with that of the synthetic pesticide permethrin 3.2 EC and aqueous DMSO (the control). Compared with the control, treatment with the bottle gourd leaf extract dramatically reduced looper larval infestation in field-grown cabbage by 41.18%, 39.71%, 52.08%, and 37.96% at the fifth, sixth, seventh, and eighth weeks, respectively. The bottle gourd leaf extract exhibited strong biopesticide efficacy against cabbage looper, increased the cabbage yield by 15.98% compared with that of the control group, and approached the yield of cabbage plants treated with Permethrin 3.2 EC.

Twenty-one compounds were identified from the ethanolic extract of bottle gourd leaves by LC–MS analysis. Compared with other components, there are greater amounts of apigenin-7-O-glucoside (2,2-dichloro-1-(4-methyl-benzoylamino)-vinyl)-phosphonic acid diethyl ester; 2-amino-9H-pyrido[2,3-b]indole; indolebutyric acid; phenol, 2-methoxy-4-(1-propenyl)-, acetate, (Z)-; phytol; 7-acetyl-1,3-dimethylpurine-2,6-dione; and (1Z)−1-[(3,4-diethoxyphenyl)methylidene]−6,7-diethoxy-3,4-dihydro-2H-isoquinoline and hexadecanoic acid. Among those compounds, apigenin-7-O-glucoside, a flavonoid, has been shown to have insecticidal effects on insects. It can also be used as a starting point for the development of new bioinsecticides. Apigenin-7-O-glucoside can kill insects and reduce their ability to lay eggs, making plants less appealing to insects, reducing their feeding and growth, and making it harder for insects to digest plants, thus reducing their survival [35, 36]. Indole-3-butyric acid (IBA) has been used in combination with other chemicals to increase the resistance of plants to insects [37]. Strychnine is a pesticide used to control insects, rodents, and birds. It is also known as a rodenticide, an avicide, or an insecticide [38]. Phytol shows considerable potential for use in ecofriendly green insecticides [39]. Hexadecanoic acid has nematicide and pesticide activities [40]. The presence of primary aliphatic amines, carboxylic acids, alkanes, alcohols, sulfones, phenols, primary alcohols, anhydrides, 1,2,4-disubstituted, alkenes, and halo compounds was validated by the FTIR analysis presented in Table S3. The present findings showed that the bottle gourd leaf extract functions as an effective insecticide against cabbage looper larvae. In future research, it will be useful to further improve the formulation of the bottle gourd leaf extract for practical applications to control cabbage looper crop infestations under field and laboratory conditions.

## Conclusion

Compared with the control and Permethrin 3.2 EC treatments, treatment with the bottle gourd leaf extract reduced cabbage looper infestation and improved cabbage productivity. Apigenin-7-O-glucoside, indole-3-butyric acid, strychnine, phytol and hexadecanoic acid are target compounds identified in the extract. Bottle gourd leaf extract can be used as an eco-friendly biopesticide instead of as a toxic chemical pesticide to control cabbage looper in a sustainable way. Because the current study was a small-scale field trial, additional research is needed to assess the potential practical use of bottle gourd leaves as biopesticides.

## Data Availability

All the data generated during this study are included within the article.
